# Promoting the bio-cathode formation of a constructed wetland-microbial fuel cell by using powder activated carbon modified alum sludge in anode chamber

**DOI:** 10.1038/srep26514

**Published:** 2016-05-20

**Authors:** Lei Xu, Yaqian Zhao, Liam Doherty, Yuansheng Hu, Xiaodi Hao

**Affiliations:** 1 UCD Dooge Centre for Water Resource Research, School of Civil Engineering, University College Dublin, Belfield, Dublin 4, Ireland; 2 Key Laboratory of Subsurface Hydrology & Ecology in Arid Areas (Ministry of Education), School of Environmental Science & Engineering, Chang’an University, Xi’an 710054, China; 3 Beijing University of Civil Engineering and Architecture/Beijing Climate Change Research and Education Centre, Beijing 100044, PR China

## Abstract

MFC centered hybrid technologies have attracted attention during the last few years due to their compatibility and dual advantages of energy recovery and wastewater treatment. In this study, a MFC was integrated into a dewatered alum sludge (DAS)- based vertical upflow constructed wetland (CW). Powder activate carbon (PAC) was used in the anode area in varied percentage with DAS to explore its influences on the performance of the CW-MFC system. The trial has demonstrated that the inclusion of PAC improved the removal efficiencies of COD, TN and RP. More significantly, increasing the proportion of PAC from 2% to 10% can significantly enhance the maximum power densities from 36.58 mW/m^2^ to 87.79 mW/m^2^. The induced favorable environment for bio-cathode formation might be the main reason for this improvement since the content of total extracellular polymeric substances (TEPS) of the substrate in the cathode area almost doubled (from 44.59 μg/g wet sludge to 87.70 μg/g wet sludge) as the percentage of PAC increased to 10%. This work provides another potential usage of PAC in CW-MFCs with a higher wastewater treatment efficiency and energy recovery.

Natural resources for freshwater production and energy generation are depleting at an unprecedented rate. It was estimated that two-thirds of the global population will face water quality problems by 2025 while the demand for water consumption will increase more than 40% by 2050^1^. Beyond this, primary energy consumption will also increase by 37% between 2013 and 2035^2^, implying that 4.5 billion TOE of energy will be required to meet the balance. This severe situation has prompted research into developing sustainable technical platforms for water recycling and renewable energy production. The appearance of microbial fuel cell (MFC) technology, which is capable of extracting energy during wastewater treatment, highlights the potential to cope with either challenge with a small environmental footprint.

Most recently, a new hybrid technology based on the principle of MFCs was developed by embedding MFCs into constructed wetlands (CWs), giving the name CW-MFCs^3^,^4^,^5^,^6^. CWs have been well recognized and increasingly applied worldwide as an environmentally friendly technology for the treatment of various wastewaters. With this integration, CW-MFCs can directly extract “waste energy” (organic pollutants) from wastewater with simultaneous water purification, with the possibility of becoming a net energy producer if most of the potential energy contained in the wastewater can be harnessed^7^.

Significant improvements of pure MFCs were achieved during last several years, with the highest power density reaching as high as 2870 W/m^3^ under certain circumstances^8^. However, this value is usually lower than 30 W/m^3^ when the pure MFC’s volume increases to over 2 L^9^. In terms of CW-MFCs, the maximum power density is less than 50 mW/(wetland area m^2^) at present^6^ due to lower columbic efficiency or higher power loss (activation, mass transfer and ohmic loss etc.) from the integration of MFC in a comparably larger volume CW. Latest studies have attempted to change the flow regimes^10^ or optimize the cathode materials and configurations^11^ to improve the system output. However, the value is still restricted. Undoubtedly, one of the priorities of CW-MFC development is to enhance its power generation.

Powder activate carbon (PAC), a well-known cost-effective material with high specific area, has been widely applied as an adsorbent in various wastewater treatment processes for different pollutants removal. It has also been used to provide sufficient adhesive surface for microorganism growth, while these attached bacterial can also utilize the adsorbed and/or surrounding organic pollutants to maintain their metabolism^12^,^13^. In terms of its application in MFC systems, owing to its electrical conductivity and comparable catalytic performance compared to platinum (Pt) in oxygen reduction reactions^14^,^15^, most of the studies were focused on the activated carbon modified air-cathode MFCs^16^,^17^,^18^,^19^. It can effectively improve oxygen reduction efficiency on cathode surface, thus enhancing the performance of MFCs. In addition, some studies are using granular activated carbon as a capacitive bioanode and then recirculating them in the anode chamber of a fluidized MFC system, which provides a new concept to broaden the practical use of MFC systems^20^,^21^.

In this study, five CW-MFC systems (four for testing and one for control) were set up, which employed dewatered alum sludge (DAS) as the wetland substrate while PAC was adopted to modify the DAS in the anode area to explore the enhanced performance on electricity generation and wastewater treatment. Emphasis was placed on the role of PAC in reducing the internal resistance (activation and ohmic losses). The influences of the percentage of PAC versus DAS on the performance of the CW-MFC were examined under continuous operation of the system in vertical flow mode.

## Result and Discussion

### Influence of PAC additions to CW-MFC effluent quality

Diluted swine water with the designed concentration was continuously fed to five parallel CW-MFC systems. Effluent quality and the related parameters of each system were examined throughout the stable operation period (about 4 months) and the results are shown in Table 1. Compared to these operated under open-circuit condition (Table S1), it is clearly to see that the integration of MFC into CWs further enhanced the removal of COD, but no significant differences were observed in terms of RP, NO_3_-N and NO_2_-N. However, it is interesting to find out that the integration slightly improved the NH_4_-N and TN removal, which means the improvements in both nitrification and denitrification process since NO_3_-N in the effluent didn’t show the corresponding changes. Many previous studies have showed the possibility by using MFC for nitrogen removal through SND (simultaneously nitrification and denitrification) process^22^. This can directly contribute to the removal of nitrogen. Other aspects like electrical stimulation^23^ or denitrification bio-cathode formation^24^ might also result in the improvements on denitrification process.

More specifically to those operated under close circuit, COD was significantly removed in all systems. Based on our previous studies^4^,^10^, DAS based CW-MFC can effectively remove COD in swine wastewater with concentration ranging from several hundreds to thousands mg/L. In this study, the control system without PAC modification shows a similar removal efficiency of COD (around 70%) compared to our previous studies. Moreover, it is clearly demonstrated that DAS/PAC substrate can enhance COD removal up to more than 80% with 10% of PAC addition. This is mainly the result of the naturally superior adsorption ability of PAC and its ability to form Bio-PAC as time goes by, which contributed to the removal of organic matter in the influent^25^. In addition, the integration of MFC in CWs can also contribute to enhanced removal of COD^26^,^27^ as referred above.

With regard to phosphate removal, it has already been shown that DAS has excellent phosphate adsorption ability due to the strong adsorption affinity between Al in DAS and P in the influent^28^,^29^. The adsorption is mainly governed by ligands exchange processes^30^. With the amendment of PAC (10% addition), the removal efficiency of reactive phosphate (RP) in the CW-MFC system was further increased to nearly 90%. Physical adsorption on activated carbon is likely the main mechanism for the enhancement of phosphate removal^31^,^32^.

In terms of nitrogen removal, ammonia-N removal efficiency of the control system is lower than 40% (from 26.40 ± 4.2 mg/L to 15.45 ± 3.3 mg/L). This is mainly due to the insufficient oxygen provision for the nitrification process, since without an artificial aeration system, nitrification can only happen near the surface of the system where the oxygen dissolved form air can be utilized (with DO of around 2.0 mg/L). It is also noted in Table 1 that along with the removal of ammonia-N, nitrate-N in the effluent is significantly increased. However, its amount is obviously less than the decrease of ammonia-N, implying that denitrification occurred in the systems. The occurrence of denitrification could be attributed from two aspects: 1) as indicated by Vazquez *et al*.^33^, though the upper region of CW remained aerobic condition at the macro-scale, anaerobic and anoxic conditions can occur at the micro-scale. The accumulation of organic matter on the surface of substrate around the bio-film can limit oxygen diffusion into biofilm promoting the denitrification process. 2) Another potential reason might be the existence of aerobic denitrifiers developed on the top of the system as reported by Austin *et al*.^34^, which is capable to use either nitrate or oxygen as a terminal electron acceptor, resulting into the denitrification process occurred under the aerobic surroundings^35^,^36^. Both of which can result in the decrease of total nitrogen (TN) during the whole operation period (Table 1). In comparison with those systems of PAC addition/modification, it is clear that the addition of PAC enhanced TN and ammonia-N removal efficiency. As aforementioned, PAC has advantages in substrate adsorption, such as phenols and polynuclear aromatic hydrocarbons etc^37^,^38^. Both of which are toxic and typical nitrification inhibitors that reduce ammonia-N removal^39^,^40^, resulting in lower TN removal. Therefore, with the additions of PAC in the anode compartment, related toxic substances can be significantly removed which contribute to the higher ammonia-N and TN removal efficiencies (increasing of 11.3% and 12.2%, respectively), and the higher ammonia-N removal corresponding to an increase of nitrate-N in the effluent. It should be pointed out that no significant nitrite-N was detected during the whole period. This can be caused by the reasons that: 1) simultaneous nitrification denitrification (SND) process occurred in the system as presented by several previous studies^36^,^41^; 2) the existence of *Nitrobacter* can directly oxidize nitrite into nitrate owing to the sufficient DO provision in the upper layer of the CW-MFC system (NO_2_^−^+1/2O_2_ → NO_3_^−^+ΔG^0^, ΔG^0^ = −65 ~ −90 kJ/mol)^42^. Both of which contribute to the reduction of nitrite in the effluent. More detailed studies about the influence of MFC integration on nitrogen removal will be included in our future investigation, which includes the potential of using nitrate-N as a cathode electron acceptor during the autotropic denitrification process^43^.

### Comparison of LOI and TEPS in substrate

**Figure 1:**
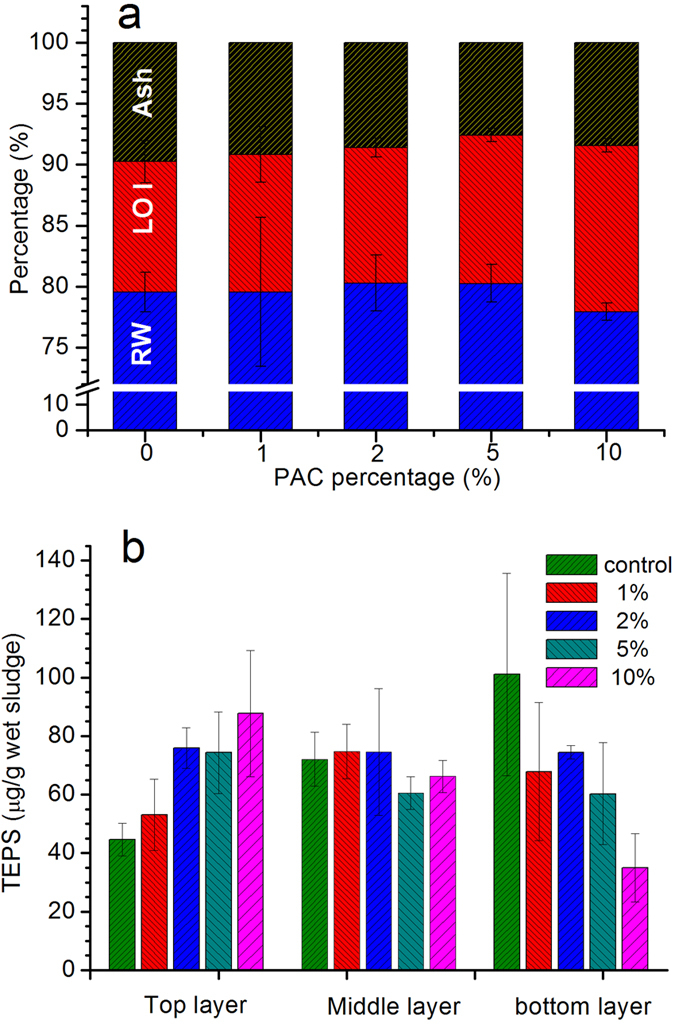
LOI (**a**) and TEPS (**b**) content in various zones of reactors with different percentage of PAC additions.

At the end of the trial, 9 substrate samples from the top, middle and bottom layers of each CW-MFC system were sampled and the LOI of each sample was measured. Differences in LOI caused by different PAC additions can be observed from Fig. 1A. Along with the increasing percentage of PAC, the LOI of the substrates increases from 10.7% to 13.6%, while the residual water (RW, %) in the substrates decreases from about 80.0% to 77.9%. The reduction in RW is probably due to the lower hydrophilic ability of PAC compared to DAS. In terms of the increasing amount of LOI, it reveals that more organic matter can be adsorbed or accumulated on PAC modified substrate compared to DAS, which directly contributes to the enhanced COD removal as the content of PAC increased (Table 1). Since more organic matter adsorption means more fixed biodegradable dissolved organic matter in the substrate, which might be utilized by microorganisms forming a Bio-PAC when PAC was added to the CW-MFC system.

In order to explore the impact of PAC additions to the colonization of microbes in the system, TEPS of the substrates taken from top to bottom of each system were measured. It should be clear that more microbes refer to more secretion of metabolites to extracellular space, resulting in a higher concentration of EPS, which mainly consists of polysaccharide and protein^44^. Comparing the TEPS content in different regions of each system (Fig. 1B), it is interesting to find that different changing trends in different regions were stimulated by the percentage of PAC added. At the upper part of the system (i.e. around the cathode chamber), the amount of TEPS shows a significant increase from 44.59 μg/g wet sludge to 87.70 μg/g while the content of TEPS in the middle layer of the system fluctuated between 60.54 μg/g and 74.73 μg/g. However, in terms of the bottom area, the concentration of TEPS sharply decreased from 101.03 μg/g to less than 35.00 μg/g when the percentage of PAC increased from 0% to 10%. This is different from the previous study on PAC dosage during the ultrafiltration process, where the PAC additions acted as a supporter for microorganisms and the adsorbed dissolved organic matter facilitated their propagation^44^. The explanation to this result can be from the overview of the wastewater treated in the CW-MFC systems. Unlike the source of water for drinking water treatment, heavy metals and phenols etc. commonly exist in swine wastewater^45^,^46^, both of which can greatly influence the metabolism of microorganisms and hinder their growth. Therefore, accumulated toxic substances can negatively influence the growth of microorganisms at the bottom of the CW-MFCs. Furthermore, the amendment of DAS with PAC might also have influences on the biocompatibility of the anaerobic bacteria colonizing the DAS surface. Both of which can result in a decrease in the amount of TEPS of the bottom substrates. Conversely, the presence of PAC modified substrate layers at the bottom can remove most of those unfavorable substances through the adsorption process^47^,^48^, which favored the colonization of those microbes at the top of the system and thus facilitated the excretion of EPS.

### Effect of PAC modified substrate on power generation

#### Polarization curve and electrode potential

**Figure 2:**
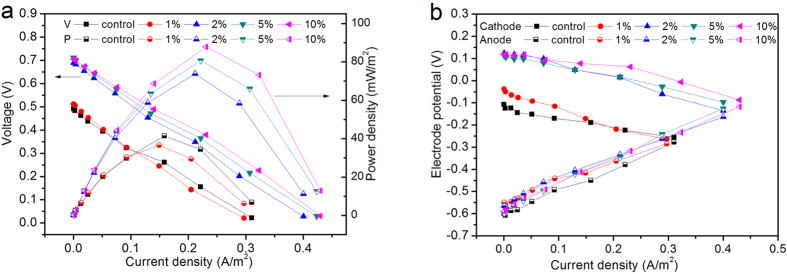
Electricity performance of CW-MFC with different percentage of PAC additions: (**a**) power density and polarization curve; (**b**) electrode potential.

The main purpose of this study lies in exploring the role of PAC addition on the electrical performance of the CW-MFC systems. To this end, polarization and power density curves were generated (Fig. 2A) at the end of the trial. It is evident that PAC has significant influence on power generation and the power output was greatly enhanced when the percentage of PAC addition into DAS reached a certain percentage. With the addition of PAC of 1%, no significant influence on the maximum power density (MPD) was found; in fact a slight decrease from 41.39 mW/m^2^ to 36.58 mW/m^2^ was observed. In contrast, when the percentage of PAC increased to 2%, MPD almost doubled (from 36.582 mW/m^2^ to 73.8 mW/m^2^). With further increase of PAC percentage to 10%, MPD increased to 87.79 mW/m^2^. From the polarization curves, it is also obvious that the open circuit voltage was significantly improved by PAC additions when its content is higher than 2% (from about 500 mV to over 700 mV).

In order to explore the reasons behind those differences, electrode potentials of each system were measured (Fig. 2B). From the electrodes potentials, it is clear that most of the differences are a reflection of the improvements on cathodes potential. While PAC additions decrease the anode potential to some extent this may not actually be favorable for the growth of electrogens in the anode compartment since a more negative potential could provide more energy for the maintenance and growth of anaerobic bacteria^49^.

Recalling the content of TEPS within each system, the addition of PAC in the anode chamber will suppress the growth of microbes which might be the main reason for the decrease of the anode potential. Some previous studies found that when granular activated carbon was used as exoelectrogenic bacteria support material, it can function as an electron capacitor. With intermittent contact of the anode electrode, the fluidized particles are more efficient than packed-bed style^20^ (47% higher current density). Though the PAC can be regarded as the biofilm matrix which provides a larger surface area for exoelectrogenic bacteria growth - compared with the restriction of the small SSM anode surface area - the biofilm at a greater distance from the SSM can contribute less to electricity generation. This might be another reason why the addition of PAC in the anode compartment didn’t show its advantages in terms of electrical output. Thus, it is reasonable to believe that the contact probability and efficiency of substrates at the anode surface are the two key issues to increase the electrons transfer efficiency in the anode compartment.

As indicated by the changing trends of the amount of TEPS in substrates taken from each reactor, PAC additions can favor the growth of microbes near the surface of the reactor, which may contribute to the formation of a bio-cathode. Unlike abiotic cathodes, bio-cathodes can use bacteria as biocatalysts to intrinsically decrease the activation losses of cathode reactions, which can facilitate the oxygen reduction reaction in air-cathode MFCs^50^. In addition, Wang *et al*.^51^ showed that compared to abiotic-cathode, biocathode can significantly decreased the charge transfer resistance between cathode and oxygen (from 174.6 Ω to 15.4 Ω), which resulted into a 14 times higher maximum power density production. Although it is still not clear whether this is a respiratory governed process or through others catalysis mechanisms^52^, the reduction processes can be completed either through direct electron transfer or indirect electron transfer using self-excreted redox-active compounds^53^. Furthermore, the excretion of EPS can also enhance the adhesive ability of microbes on the cathode surface, which could also contribute to the improved performance of the cathode.

#### Internal resistance distribution

Internal resistance can be used to indicate the amount of energy lost during electricity production. The information about the changes of the internal resistances brought by the addition of PAC will help to explore the role of PAC in CW-MFC systems. Therefore, the distribution of internal resistance in each system was evaluated. For near linear polarization, the internal resistance (R_int_) can be regarded as the sum of anodic resistance (R_a_), cathodic resistance (R_c_), membrane resistance (R_m_), and electrolyte resistance (R_e_). Here, the relationship between external voltage (E) and current (I) can be derived from the equation proposed by Fan *et al*.^54^ and simplified as:


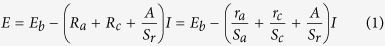


where, E_b_ (V) is the linear extrapolation open circuit voltage (LE-OCV); r (Ω m^2^) is the area-specific resistance (ASR) of respective electrode; S (m^2^) is the projected area and S_r_ is cross section area of the reactor; A is decided by the electrodes distance and electrolyte concentration.

The values of *E*_*b*_, *r*_*a*_, *r*_*c*_ and *A* are determined using the SOLVER function in Microsoft excel with a best fit of the experimental date with Eq. (2) and the results are listed in Table 2. It can be noted from Table 2 that the major internal resistance is from electrolyte resistance (from 71.22% to 85.48%), while the internal resistance arising from the anode and cathode only account for less than 30%. Both anodic and cathodic resistances are reduced as the proportion of PAC increased, while the electrolyte resistance increased. The decrease of anodic resistance is probably a result of the conductivity improvements of the substrates with PAC addition, which can lower the activation energy barrier for electron transfer between the biofilm and the anode electrode^55^. The decrement of cathodic resistance likely resulted from the improved reduction reactions due to the existence of bio-catalysts formed on the surface of the cathode electrode. For the increment of electrolyte resistance, the reduction of ions concentration (removed through PAC adsorption process) in the electrolyte should be the main reason. In consequence, the total internal resistance of each system actually shows no significant differences, through which it is reasonable to conclude that the reduction of electrode distance in CW-MFC will be the most efficient method to reduce the internal resistance. As also be revealed by Deng *et al*.^56^ both substrate and overlaying water depth can significantly influence the internal resistance of the system.

Based on the influences of PAC additions on power output of CW-MFC described above, it seems that though the amount of microorganism around the anode area decreased, the performance of the anode electrode was not influenced too much. In comparison with the anode, the performance of the cathode was greatly enhanced due to the bio-cathode formation. This reveals the fact that the anode is not the primary factor in controlling how much electricity can be generated in the CW-MFC. Taking glucose (C_6_H_12_O_6_) as an example, with 1 mol of glucose oxidation, 24 mol electrons will be released which can provides enough electrons for 6 mol of oxygen reduction. Considering the slow catalytic kinetics of oxygen reduction, the electrons produced is abundant for cathode reactions if only oxygen was considered as the electron acceptor. Thus future work should focus on the improvement of cathode performance, rather than the anode compartment. Methods such as utilizing materials to promote the catalytic property of the electrode or adopting other electron acceptors (nitrate or metal ions) warrant further investigation. Since most of the internal resistance comes from electrolyte resistance which strongly relates to the electrode spacing, reducing the spacing and keeping respective electrodes working under its favorable conditions is the most effective way to minimize the energy losses from ohmic resistance.

#### Columbia efficiency (CE) and net energy recovery (NER)

**Figure 3:**
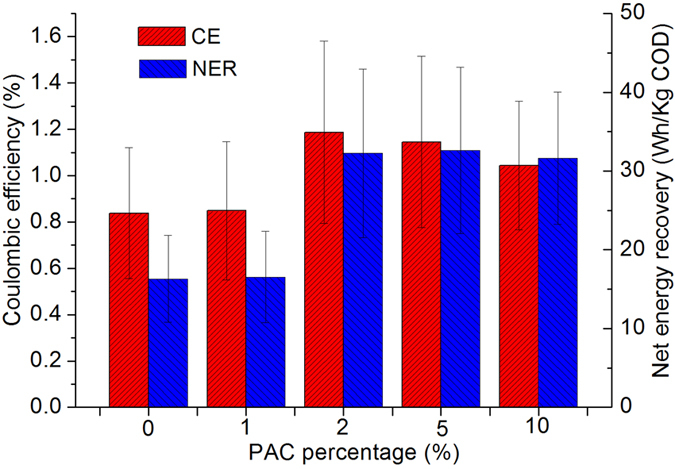
CE and NER of each system with different PAC additions.

CE is an important parameter in MFC technology since it represents the amount of organic matter converted to electrical current, which can be used to evaluate the contributions of MFCs within the hybrid systems. Here, the average CE of each system under the stable operational period was calculated (Fig. 3). It shows that all the systems present a low CE of around 1%. Among which the one with 2% of PAC shows the highest CE (1.2 ± 0.4%) compared to others, while higher PAC shows slightly lower CE to some extent. This is mainly caused by the higher adsorption of organic matter on PAC as its content increased. Thus the ratio of organic matter consumed by microbes (which could contribute to the output directly) to the whole COD removed decreased. On account of this, 2% of PAC additions would be the reasonable choice in this study. In previous studies, low CEs (lower than 4%) in CW-MFC systems are a big challenge that needs to be addressed. This reveals that although large percentages of the COD was removed through biological oxidation, the electrons released were not effectively captured or utilized on anode and cathode, respectively. Improvements of CEs in CW-MFCs or any other MFC integrated systems should focus on either electron collection at the anode or their utilization in reduction reactions at the cathode.

Another key parameter used to assess the performance of MFCs is the NER (KWh/m^3^ or KWh/Kg COD), which reflects the ability of MFC to convert organics in wastewater into electricity. Based on previous studies, the theoretical highest NER of pure MFCs is about 3.86 KW/Kg COD, which is considerably higher than most of the studies of MFCs at present (less than 1.0 KW/Kg COD)^9^. Though it was revealed by Ge *et al*.^9^ that NER seems to have no direct relationship with reactor dimension and/or power densities, larger volume reactors lead to a lower NERs due to lower coulombic efficiencies. In this study, the NER of each system under an external load of 950 Ω was examined (Fig. 3). From the data, a comparable changing tendency between CE and NER can be observed. Along with the increase of the percentage of PAC, NER increased from about 15 Wh/Kg COD to more than 30 Wh/Kg COD, doubling the electricity recovery compared to the control system, while higher percentage of PAC (>2%) did not influence the NER greatly. However, the NER is still much lower than the theoretical maximum, mainly due to the poor performance in terms of CE. Overall, based on the results of CE and NER, a low percentage of PAC cannot contribute to a better performance of the system in terms of energy recovery. When the content of PAC reaches a certain level, energy recovery efficiency can be significantly improved while further additions show little influences or even have negative influence on CE and NER. Therefore, PAC addition of 2% is the best option based on this study.

## Materials and Methods

### CW-MFC construction and inoculation

**Figure 4:**
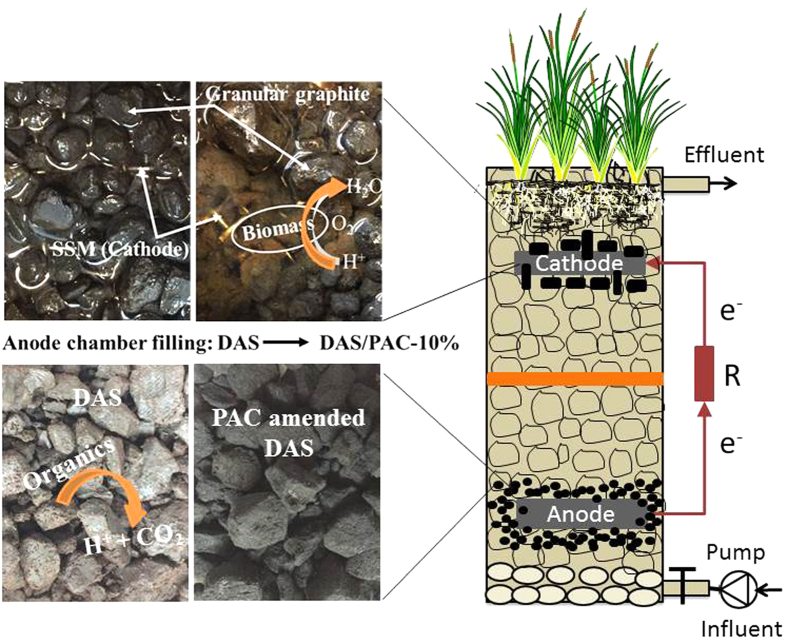
Schematic description of the CW-MFC system.

Five lab-scale vertical flow CW-MFCs, with identical dimensions (Φ 0.15 m × 0.32 m), were set up with their configuration shown in Fig. 4. DAS, collected from a local water treatment plant treating reservoir water using aluminum sulphate as coagulant, was used as the main wetland substrate in all the systems. The average particle size of the substrate/DAS was 10–15 mm while the average porosity was around 0.3, which resulted in a net liquid volume of about 1.5 L for each system. Stainless steel mesh (SSM, thickness of 1 mm, 5 Mesh) was used as both the anode and cathode of the embedded MFC with a working surface area of 70 cm^2^ and 35 cm^2^ (n × surface area, n represents the number of steel wires contained in the mesh) respectively. Among each CW-MFC, cathode and anode were buried close to the surface and bottom of the system, respectively, which resulted in an electrode spacing of about 200 mm. The cathode compartment was filled with a layer of granular graphite (GG) (diameter 8–13 mm, initial porosity of 0.38, pretreated by soaking in 0.1 M HCl for 24 h then washed 3 times with deionized water to remove its surface contaminates) and located at the air–water interface. The anode compartment consisted of the PAC modified DAS (DAS/PAC) with different percentages of PAC ranging from 1% wt (PAC/DAS ratio) to 10% wt. This configuration of CW-MFCs set up remains the same for four systems while one system had no PAC, and served as a control. Cathode and anode were connected by insulated titanium wire through an external circuit with a load of 950 Ω, which was chosen based on our previous work^10^. At the very bottom of each system, a depth of 30 mm gravel with average diameter of 5 mm was filled to improve the distribution of the wastewater in the wetland. Prior to the start-up of all systems, a period of three weeks was used for the inoculation of the anode compartment with anaerobic digestion sludge sourced from a local Wastewater Treatment Plant.

### Wastewater and operation conditions

Swine wastewater was collected weekly from a local agriculture research farm. The influent wastewater was diluted with tap water to obtain a COD concentration of about 500 mg/L, which resulted in average total nitrogen (TN), ammonium (NH_4_^+^-N) and reactive phosphorous (RP) concentrations of 40.5 ± 5.7 mg/L, 26.4 ± 4.2 mg/L and 9.8 ± 1.3 mg/L, respectively. The prepared wastewater was then continuously pumped into each CW-MFC system (from the same influent tank) at the bottom, passing through the anode compartment, cathode compartment and finally left the system from the upper outlet. Influent flow rate was controlled through peristaltic pumps, giving an average hydraulic retention time (HRT) of about 1 day for all systems. All the experiments were conducted at room temperature (15 ± 5 °C) during the whole operation period of 4 months.

### Measurements and calculation

#### Water quality

After a period of steady operation, the performance of CW-MFCs in wastewater treatment was investigated via pH, DO, COD, TN, NH_4_^+^-N, NO_3_^−^-N, NO_2_^−^-N, RP. Among them, pH was measured using a pH meter (Orion 920 A+, Thermo); DO was determined through a microprocessor oximeter (Oxi 325, WTW); COD, NH_4_^+^-N, NO_3_^−^-N, NO_2_^−^-N and RP were analysed using Hach DR/2400 spectrophotometer according to its standard operating procedures. TN was determined with persulfate method^57^. For accurate results, dual-samples were taken and monitored while the results were presented as average value with its standard deviation.

#### Loss on ignition (LOI) and total extracellular polysaccharide (TEPS) of the substrate

LOI is usually used to provide a rough approximation of the total organic matter (TOC) presented in the solid fraction of the sample^57^. TEPS can be used to reflect the amount of microorganisms in the sample^44^. For LOI, substrate samples taken from each CW-MFC system were dried overnight at 105 °C to eliminate its residual water. Thereafter, 5 g of each sample was used for the LOI tests, which were conducted in a furnace at 500 °C using a thermostatic temperature controller and the loss on weight was regarded as the TOC in the substrate. In terms of TEPS measurements, the method was similar to Yu *et al*.^44^. Substrates were sampled in centrifuge tubes and centrifuged at 3000 rpm to discard the supernatant. The sample remaining in the centrifuge tube was then suspended in 10 mL phosphate buffer saline (PBS) solution followed by 5 min ultrasonic treatment and another 5 min vigorous mixing. The mixture was then heated to 80 °C in a water bath for 30 min and finally subjected to centrifuge at 8000 rpm for 10 min. The supernatant collected at this stage was regarded as the TEPS. After the extraction process, the polysaccharide content of each sample was measured by the phenol–sulfuric acid method with glucose as the standard^58^.

#### Power generation performance

The electricity generation was monitored through either a digital multimeter or a data logger (USB-6000, National Instruments) in terms of the voltage drop (V) across the external resistor. Power densities (P = U^2^/RS, mW/m^2^) were determined through basic electrical calculations, where U is the voltage (V), R is the resistance (Ω) and S is the surface area of the cathode (m^2^). To obtain the polarization curves, external resistance was varied over a range from 580,000 Ω to 20 Ω and the steady-state voltage across the resistors was measured. The electrode potentials were determined against a saturated Ag/AgCl electrode (Mettler Toledo).

The coulombic efficiency (CE, %) (the fraction of electrons used for electricity generation versus the electrons in the starting organic matter) was calculate through the formula shown in Eq. (1), which was derived from Logan^59^ for MFCs fueled by complex wastewaters at a continuous rate.


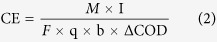


where, M is molecular mass of O_2_ (g O_2_/mol O_2_), which is 32. I is current (A) and F is Faraday’s constant (C/mol), which is 94,685. q is flow rate (L/s) while b is number of electrons donated per mole O_2_ (mol e^−^/mol O_2_), which is 4. Finally, ΔCOD represents the change in COD between influent and effluent (g/L).

## Additional Information

**How to cite this article**: Xu, L. *et al*. Promoting the bio-cathode formation of a constructed wetland-microbial fuel cell by using powder activated carbon modified alum sludge in anode chamber. *Sci. Rep*. **6**, 26514; doi: 10.1038/srep26514 (2016).

## Supplementary Material

Supplementary Information

## Figures and Tables

**Table 1:** The water quality parameters between influent and effluent with different percentage of PAC.

Parameter	Influent	Effluent with different percentage of PAC				
		Control	1%	2%	5%	10%
COD	484(63)	145(18)	142(22)	147(16)	125(19)	90(11)
TN	40.5(5.7)	27.3(2.8)	25.2(3.1)	25.0(2.6)	23.6(3.3)	22.3(2.4)
NH_4_-N	26.40(4.2)	15.45(3.3)	13.95(2.5)	13.65(2.7)	13.65(3.6)	12.45(1.9)
NO_3_-N	0.13(0.22)	3.26(0.38)	4.15(0.29)	4.41(0.44)	4.52(0.61)	5.58(0.83)
NO_2_-N	0.03(0.01)	0.11(0.09)	0.66(0.62)	0.07(0.05)	0.04(0.02)	0.06(0.04)
RP	9.8(1.3)	2.8(0.3)	1.8(0.4)	1.4(0.2)	1.4(0.2)	1.2(0.2)
DO	0.6(0.1)	2.1(0.1)^*^	2.1(0.1)^*^	1.9(0.1)^*^	2.0(0.1)^*^	2.0(0.1)^*^
pH	8.289(0.018)	6.850(0.057)^*^	7.014(0.022)^*^	7.080(0.034)^*^	6.814(0.093)^*^	7.075(0.057)^*^

Unit: mg/L except pH; () are standard deviation of each indexes; ^*^means cathode chamber measurements.

**Table 2:** Internal resistance distribution among each reactor with different PAC additions.

PAC (%)	R_a_ Ω (%)	R_c_ Ω (%)	R_e_ Ω (%)	R_int_ Ω	Fitting equation	R^2^
0	32.428 (7.68)	89.139 (21.1)	300.875 (71.22)	422.438	*E* = 0.482–422.438 *I*	0.9954
1	39.968 (9.36)	66.404 (15.55)	320.627 (75.09)	426.999	*E* = 0.462–426.999 *I*	0.9948
2	20.884 (4.50)	61.858 (13.33)	381.349 (82.17)	464.090	*E* = 0.678–464.090 *I*	0.9986
5	9.746 (2.15)	60.708(13.38)	383.29 (84.47)	453.747	*E* = 0.703–453.767 *I*	0.9985
10	10.937 (2.48)	53.04 (12.03)	376.746 (85.48)	440.723	*E* = 0.706–440.723 *I*	0.997
