# Supplementary material for: Promoting the bio-cathode formation of a constructed wetland-microbial fuel cell by using powder activated carbon modified alum sludge in anode chamber
Source: Sci Rep. 2016 May 20;6:26514. doi: 10.1038/srep26514 (PMC4873795; doi:10.1038/srep26514)
Supplement: Supplementary Information [file srep26514-s1.pdf]

Supplementary Information

Promoting the bio-cathode formation of a constructed wetland-microbial fuel cell by using powder activated carbon modified alum sludge in anode chamber

Lei Xu[1], Yaqian Zhao[1,2]*, Liam Doherty[1], Yuansheng Hu[3] & Xiaodi Hao[3]

[1]UCD Dooge Centre for Water Resource Research, School of Civil Engineering, University College Dublin, Belfield, Dublin 4, Ireland

[2]Key Laboratory of Subsurface Hydrology & Ecology in Arid Areas (Ministry of Education), School of Environmental Science & Engineering, Chang'an University, Xi'an 710054, China

[3]Beijing University of Civil Engineering and Architecture/Beijing Climate Change Research and Education Centre, Beijing 100044, PR China

*Corresponding author (Yaqian Zhao: yaqian.zhao@ucd.ie)

Supplementary Table S1. The water quality parameters between influent and effluent with different percentage of PAC under open-circuit

Parameter	Influent	Effluent with different percentage of PAC				
		control	1%	2%	5%	10%
COD	491(75)	156(12)	152(18)	155(13)	139(11)	113(8)
TN	41.3(4.9)	28.9(2.1)	26.9(1.7)	26.6(1.9)	26.1(2.6)	25.2(3.1)
NH_4-N	27.30(5.2)	16.59(1.9)	15.02(1.2)	14.83(2.1)	14.92(1.6)	14.15(1.1)
NO_3-N	0.07(0.02)	3.51(0.21)	4.28(0.18)	4.39(0.33)	4.61(0.26)	5.66(0.41)
NO_2-N	0.03(0.01)	0.09(0.05)	0.38(0.31)	0.07(0.04)	0.06(0.02)	0.07(0.03)
RP	10.2(0.7)	2.9(0.5)	2.1(0.2)	1.6(0.1)	1.4(0.1)	1.3(0.1)
DO	0.3(0.1)	2.2(0.1)*	1.9(0.1)*	2.1(0.1)*	2.0(0.1)*	1.9(0.1)*
pH	7.938(0.033)	6.678(0.029)*	6.916(0.038)*	6.883(0.017)*	6.696(0.047)*	6.925(0.018)*

Unit: mg/L except pH; () are standard deviation of each indexes; * means cathode chamber measurements.
